# Auto Sizing of CANDU Nuclear Reactor Fuel Channel Flaws from UT Scans

**DOI:** 10.3390/s23083907

**Published:** 2023-04-12

**Authors:** Issam Hammad, Matthew Poloni, Andrew Isherwood, Ryan Simpson

**Affiliations:** 1 The Department of Engineering Mathematics and Internetworking, Dalhousie University, Halifax, NS B3H 4R2, Canada; 2 The Inspection Analysis Department, Ontario Power Generation (OPG), 777 Brock Rd., Pickering, ON L1W 4A7, Canada; 3 The Engineering Department, Alithya Digital Technology, 1420 Bayly St., Pickering, ON L1W 3R3, Canada

**Keywords:** CANDU, fuel channel inspection, inspection automation, linear regression, non-destructive examination (NDE), nuclear reactor, pipe inspection, ultrasonic testing (UT)

## Abstract

The inspection of nuclear power plants is an essential process that occurs during plant outages. During this process, various systems are inspected, including the reactor’s fuel channels to ensure that they are safe and reliable for the plant’s operation. The inspection of Canada Deuterium Uranium (CANDU^®^) reactor pressure tubes, which are the core component of the fuel channels and house the reactor fuel bundles, is performed using Ultrasonic Testing (UT). Based on the current process that is followed by Canadian nuclear operators, the UT scans are manually examined by analysts to locate, measure, and characterize pressure tube flaws. This paper proposes solutions for the auto-detection and sizing of pressure tube flaws using two deterministic algorithms, the first uses segmented linear regression, while the second uses the average time of flight (ToF) within ±σ of µ. When compared against a manual analysis stream, the linear regression algorithm and the average ToF achieved an average depth difference of 0.0180 mm and 0.0206 mm, respectively. These results are very close to the depth difference of 0.0156 mm when comparing two manual streams. Therefore, the proposed algorithms can be adopted in production, which can lead to significant cost savings in terms of time and labor.

## 1. Introduction

**Figure 1:**
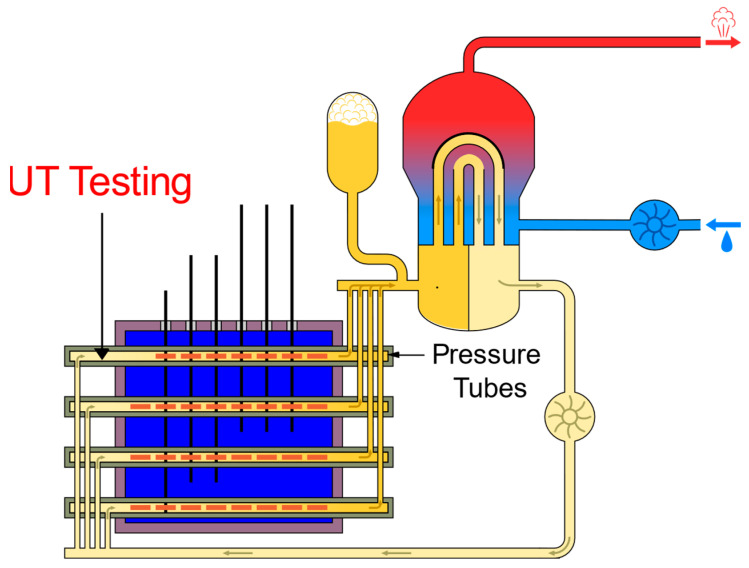
High-level design for the CANDU reactor core and the fuel channels.

A Canada Deuterium Uranium (CANDU^®^) nuclear reactor is a Canadian reactor that uses pressurized heavy water to produce electricity [1,2,3,4,5,6,7,8,9]. To ensure the safety and reliability of the reactor for operation, various systems, including the fuel channels, are continually inspected. The pressure tubes, which are core components as part of the fuel channels, must be inspected to locate and characterize flaws that may have developed over time. To determine whether a fuel channel is fit for service, ultrasonic testing (UT), a non-destructive examination (NDE) method, is used to inspect the pressure tube. Using NDE techniques for nuclear power plant inspection has been previously discussed [10,11,12,13,14,15,16]. For the inspection of pressure tubes, volumetric and surface NDE examinations are utilized [17]. As per CANDU design, each reactor contains between 380–480 fuel channels. Each fuel channel consists of a 104-mm diameter (ID), 4.3-mm thick Zirconium containing an inner Niobium pressure tube and an outer calandria tube. Additionally, it contains two stainless steel end fittings. The tubes are approximately 6.3 m long [17]. Pressure tubes contain the fuel bundles and the heavy water primary coolant. The calandria tube’s role is to keep the heavy water moderator away from the pressure tubes. Figure 1 depicts a high-level design for the reactor core and the fuel channels. During an inspection, a remotely operated tool is used to deliver NDE inspection tools into the defueled channels for inspection. The inspection tools are axially driven along the fuel channel as they rotate, resulting in a helical collection of UT data for each fuel channel. The acquired UT data (B-scans) can be represented as three-dimensional UT data with the following axes: ultrasound travel time (*X*-axis), transducer displacement (*Y*-axis), and signal amplitude (shading). This collection of data is examined afterward through manual visualization by a highly trained and certified analyst who is required to have CAN/CGSB-48.9712-2014/ISO 9712:2012 certification. Such analysis can reveal different flaw types that have developed, such as crevice corrosion, debris fretting, and fuel bundle bearing pad fretting (FBBPF).

Debris fretting is a service-induced flaw formation mechanism that occurs when entrained debris particles become lodged within the fuel string, and wear into the inside surface of the pressure tube surface under the influence of flow-induced vibration. The resultant debris fretting flaws pose a concern for PT structural integrity because they can function as preferential locations for crack initiation. The sources of debris in fuel channels are primarily construction/commissioning related, such as foreign material inadvertently left in the heat transport system during construction (e.g., welding electrodes, spatter, etc.), failed components (e.g., gaskets and fuel channel commissioning strainers), and pressure tube swarf (from initial dry fuel loading). The majority of debris fretting flaws observed to date have been characterized as localized, blunt, and shallow (<0.5 mm deep), with low-stress concentrations, but they often exhibit multi-faceted features and complex, irregular shapes. These flaws may be found anywhere along the length of the pressure tube, but they tend to be more common at the inlet bundle location and primarily affect the bottom half of the pressure tube circumference.

Crevice corrosion (CC) flaws are caused when the bearing pad of a fuel bundle does not achieve perfect contact with the inner surface of the pressure tube (PT), which forms a crevice where coolant flow is impeded. The imperfect contact restricts the heat transfer between the bearing pad and the PT, causing the “stagnant” coolant to boil, which increases the local concentration of lithium hydroxide (which is used to control the pH of the primary coolant). Under these conditions, the corrosion rate of the zirconium PT in the crevice is accelerated, which produces a crevice corrosion flaw. Crevice corrosion flaws are typically about 3 mm wide, with axial lengths from 3 mm to 12 mm, and maximum depths of ~0.5 mm.

Fuel bundle bearing pad fretting (FBBPF) flaws form as a result of the fuel bundle bearing pad wearing the PT surface, due to bundle vibration induced by PHTS flow turbulence and acoustic pulsation. The shape of FBBPF flaws generally resembles that of the rectangular bearing pads (~31 mm long by ~2.7 mm wide). Depending on the location of the fuel bundle and its support configuration, the length of the FBBPF flaws can be anywhere from 0 to 31 mm. FBBPF flaw widths are typically 2.7–3 mm. They may appear as individual isolated flaws or a cluster of multiple overlapping flaws.

One of the main challenges of the manual inspection is the existence of mechanical noise (chatter) in the UT data which has resulted from a recent inspection tooling modification. The time needed to analyze the UT data has increased as a result. The duration of outages and all related operational costs can be decreased by developing solutions that can automate the analysis of the pressure tube inspection data.

Previously, a deep-learning [18,19] solution for the detection of flaws in CANDU pressure tubes in UT scans was proposed [20]. The proposed solution was able to locate flaw regions in each UT scan with minimal false positives. The proposed solution was a binary classifier, so it was able to identify the location of flaws, but not measure their depth.

This paper proposes two deterministic algorithms to automate the process of locating and sizing pressure tube flaws in terms of depth. The first method uses segmented linear regression, while the other method uses the average time-of-flight (ToF) for the data between ±σ (one standard deviation). These automation methods allow for a reduction in the time and cost to analyze UT inspection. A reduction in analysis time is being sought by nuclear operators to address increasingly aggressive outage targets, resource pool shrinkage, and increased volumes of inspection data.

Depth sizing of flaws could be an NDE application for various piping/tubing configurations over a wide range of industrial applications. The technique described in this paper is, however, CANDU specific, as this is the only reactor design that uses Zr-Nb fuel channel pressure tubes with D_2_O primary heat transport coolant.

Unlike the previously proposed deep learning solution, which is essentially a binary classifier to determine if a flaw exists or not at a particular location with a resolution of 1 mm/2°, this proposed algorithm provides an exact prediction for the depth of each point in a UT scan. The proposed algorithms can calculate depth with a resolution of 0.1 mm/0.2° as it calculates the depth of each single A-Scan.

This paper is structured as follows: Section 2 provides background on the materials and methods used for the inspection process. Section 3 presents a discussion on the presented problem, including the used testing environment. Section 4 presents the two proposed deterministic auto-sizing methods and includes a comparison with the previously proposed computer vision method. Section 5 presents the accuracy results for the proposed algorithms, while Section 6 includes the research conclusion.

## 2. Materials and Methods

**Figure 2:**
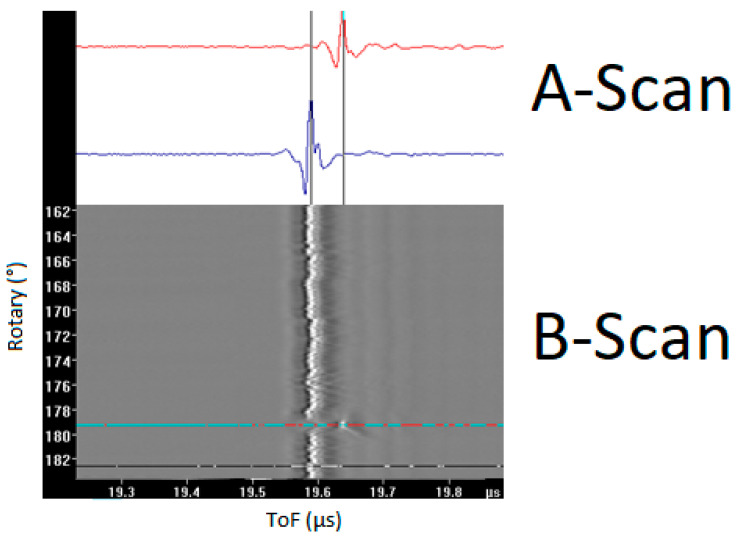
A sample for B-Scan representing A-Scan data plotted against a single coordinate variable.

The primary objective of this research was to explore the effectiveness of a deterministic auto-sizing mechanism for pressure tube UT scans by automating the analysis of Type-A and Type-D B-Scan files using the 20 MHz normal beam (NB) probe. The difference between Type-A and Type-D files is that the Type-A file contains non-interlaced data using a single probe, while the Type-D file contains interlaced data from two probes. B-Scans represent a series of A-Scan data plotted against a single coordinate variable, as can be seen in Figure 2. A-Scans can be defined as a two-dimensional graphical representation of UT data, with ultrasound travel time as one axis, and signal amplitude as the second axis.

The application described in the paper utilizes conventional (single-element) pulse-echo normal beam UT. The selection of technique and probe parameters is based on multiple factors. Probe frequencies were selected to provide a balanced compromise between resolution and attenuation. For optimal resolution, higher frequencies are needed to generate relatively short-wave lengths in the inspection medium (heavy water, in this case), but not so high as to result in significant attenuation in the inspection medium or signal cables. For normal beam depth sizing, 20 MHz was found to offer a reasonable compromise between these competing objectives. Highly damped probes are used in order to provide short ultrasonic pulses with well-defined waveform features, which can be selected for sizing measurements. The transducer case length provides a balance between being long enough to ensure sufficiently heavy damping, and short enough to allow for installation within the limited space within the inspection head ultrasonic probe module. The 20 MHz normal beam inner diameter (ID) focused probes have a nominal focal length of 10 ± 1 mm. With a nominal water path of 10 mm to the pressure tube ID surface and a focal spot beam diameter of ~0.4–0.5 mm at −6dB, this provides acceptable sizing resolution for ID flaws. The selection of the probe aperture diameter is driven by focusing requirements, signal-to-noise considerations, and space constraints in the inspection head. The natural focal length and focusing factor for a given acoustic lens is determined by the diameter, in combination with the frequency, so the range of suitable diameters for the required focal length will be constrained by the choice of frequency. Probes with a smaller diameter will tend to have lower signal amplitude and be less tightly focused, and probes with a larger diameter can be susceptible to interfering acoustic responses (noise) due to the curved surfaces of the pressure tube and probe lens. The selected aperture diameter of 6.3 mm represents a balance between sensitivity, focusing performance, and signal-to-noise ratio within the mounting space limitations imposed by the size of the inspection head and pressure tube.

This application utilizes relatively high frequency (center at −6 dB: 20+/−2 MHz), immersion-type, single-element, circular (6.3 mm), spherically focused (nominal focal length of 10 ± 1 mm), and heavy-damped (bandwidth 50% or higher) transducers. The probes are rated for temperatures up to 50 °C, and for radiation exposure up to 200 MRad.

## 3. Discussion

The current depth measurement mechanism relies on using visualization software to manually select the A-Scan with the largest time of flight (ToF) and then select a reference A-Scan for comparison. The difference in ToF between both A-Scans is obtained by comparing the time of flight at the peak amplitude of the ultrasonic reflection corresponding to the pressure tube wall interfaces. This translates to depth using pre-defined formulas. Linear interpolation can also be used by selecting two reference A-Scans.

**Figure 3:**
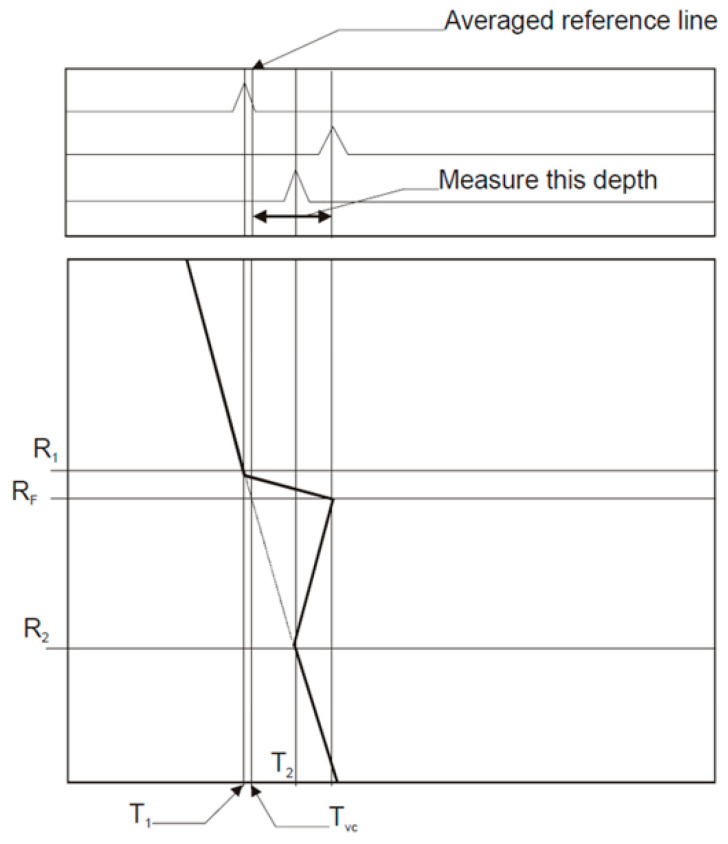
The manual depth measurement process using A-Scans.

Figure 3 demonstrates the current manual depth measurement process. Using this process, analysts establish and calculate the parameters R_1_, R_2_, R_F,_ T_1_, T_2_, and T**_vc_** as follows:

- R_1_ corresponds to the rotary position of the intersection point between the 1st horizontal cursor and the Pressure Tube (PT) Inner diameter (ID) interface signal.
- T_1_ is the time coordinate, in µs, corresponding to the R_1_ intersection point.
- The “1” subscript denotes one of two points with the lower time coordinate.
- R_2_ corresponds to the rotary position of the intersection point between the 2nd horizontal cursor and the Pressure Tube (PT) Inner diameter (ID) interface signal.
- T_2_ is the time coordinate, in µs, corresponding to the R_2_ intersection point.
- The “2” subscript denotes one of two points with the higher time coordinate.
- RF is the rotary position of the deepest point in the flaw.
- T_VC_ corresponds to the calculated location of the vertical cursor on the time axis (in us) using the formula $T_{\mathrm{VC}}=T_{2}\left[\left(R_{2}-\mathrm{RF}\right)*\frac{T_{2}-T_{1}}{R_{2}-R_{1}}\right]$.

As per the current process, two independent analyses are performed by two analysts using the same data. These are labeled as ‘Stream A’ and ‘Stream B’. Any discrepancies are later resolved by a third analyst who determines the final inspection results.

**Figure 4:**
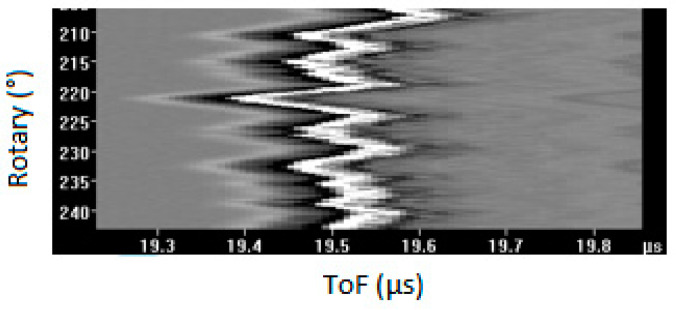
A sample for B-Scan with chatter.

As mentioned earlier, a recent modification in the inspection tooling has resulted in the introduction of unwanted mechanical noise or chatter into the B-Scan. This further increases the amount of time required by analysts to identify and size flaws. Figure 4 demonstrates an example of a case where chatter exists.

**Figure 5:**
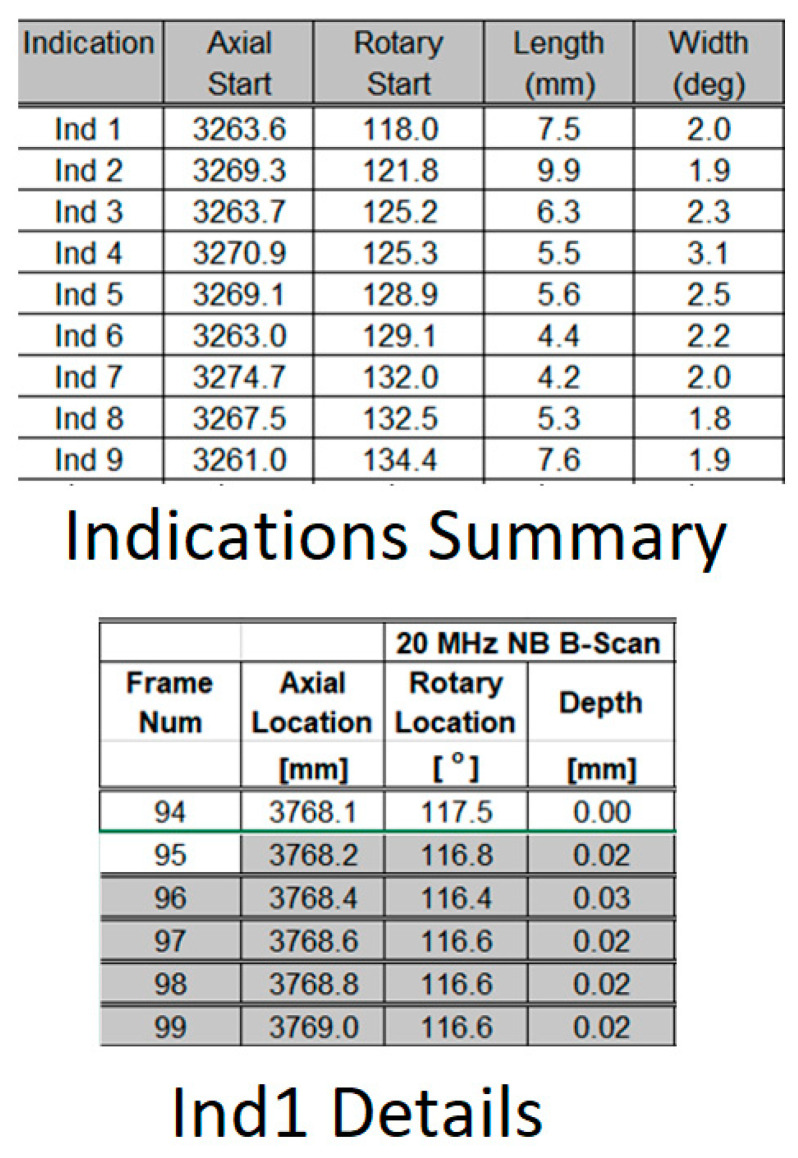
Flaw indication reporting sample (results report from one stream).

To evaluate the accuracy of the proposed auto-sizing algorithm, a testing environment was developed. This testing environment included developing python functions that can parse the B-Scan data and the corresponding previous inspection results, which are contained in the form of Microsoft Excel worksheets. This allows for automatically evaluating any proposed depth measurement algorithm against an entire outage’s inspection data, which can contain hundreds of indications (IND) representing different flaw types and sizes. For each flaw, the previous analysis results from both Stream A and Stream B were mapped. Figure 5 shows an example of sample reporting using Microsoft Excel sheets that is used by the analysts to report their findings. As can be seen, the summary report includes the axial start, rotary start, length, and width of each indication in one Excel sheet and the detailed report for each indication in another Excel sheet. This includes the exact depth of each A-Scan based on a specific axial and rotary location.

To build a testing benchmark, the performance of any auto-depth measurement algorithm would be based on the depth measurement difference (delta Δ) between the results of the first analyst (Stream A) and the results of the second analyst (Stream B). This is because each stream represents a subjective analysis of the UT data by a single analyst. Therefore, it is not realistic to expect the algorithm to produce results that exactly mimic the subjective results of one of the analysts. Given that the manual process for measuring flaw depth can be subjective, slight variation in the results is expected between ‘Stream A’ and ‘Stream B’. As an example, there might be slight differences in the selection of a reference A-Scan.

Automating the current exact manual method would be inefficient since it is difficult to develop an algorithm that can appropriately determine one or two reference A-Scans, especially in the presence of mechanical chatter. In terms of research, the real problem is determining the reference A-Scan that can be used in the depth measurement. Finding the deepest point (largest ToF) is not a hard programmatic task. The next section will present the two proposed algorithms that can be used to automate the depth measurement of an indication contained in a B-Scan.

## 4. Proposed Algorithms

To implement auto-sizing for the pressure tube defects, two algorithms were proposed, one using segmented linear regression with the other using the ToF mean for the data between ±σ (one standard deviation). For development purposes, Python was used, including NumPy [21] and sci-kit-learn [22] libraries.

The first algorithm uses segmented linear regression to build a linear model using the rotary position and the ToF for the A-Scans in the region of interest within a B-Scan frame. In general, linear regression attempts to model the relationship between two variables by fitting a linear equation. In the case of obtaining the depth measurement for the region of interest within the B-Scan, the models fit a function that consists of rotary position (x) and the ToF (y) for a specific region within a B-Scan frame. Then, the difference in ToF between the observed value and the value that is obtained using the constructed linear function will be translated into depth. This will provide the depth for each A-Scan in the region of interest. As explained in Section 3, the region of interest represents an area in a B-Scan frame representing the IND location ± the width (rotary), as specified in the channel indications summary report. The process is repeated for each B-Scan frame that contains an IND. A linear regression line can be expressed using the formula in (1), where *Y* is the response or dependent variable where, in this case, it presents the predicted depth for an A-Scan, while *X* is the predictor or independent variable where, in that case, it presents the translated ToF into depth by multiplying it with a constant for the points in the region of interest. More details about linear regression theory can be found in [23].

(1) Y=a∗X+b

For this target application, *Y* presents the expected ToF based on the peak-amplitude for the A-Scan, *X* represents the frame id within the region of interest, *a* represents the slope, and *b* represents the intercept (bias). Equation (1) can be written as:

(2) $$\hat{\mathrm{ToF}}=a*\left(frame_{id}\right)+b$$

Equation (2) is separately applied for each region of interest (IND location ± width). Therefore, for each region of interest, there is a corresponding linear regression formula. This is due to the nature of the scanned data, especially with the existence of the mechanical chatter. Each regression formula only represents a segment of the scanned region of the pressure tube. The parameters *a* and *b* are obtained for each region of interest by utilizing gradient descent [22] with mean square error (MSE) loss function, which is described in (3). For each region of interest, the function was cross-validated using k-fold cross-validation with k = 5 using 75/25 split.

(3) $$\mathrm{MSE}=\frac{1}{n}\overset{N}{\underset{i=1}{\sum }}{\left(\mathrm{ToF}i-\hat{\mathrm{ToF}i}\right)}^{2}$$

Finally, the predicted depth for each A-Scan is calculated by multiplying the predicted ToF with a constant *c*. The constant is pre-defined based on the UT scanning and calibration settings using (4).

(4) $$\hat{Depth}=\hat{\mathrm{ToF}i}*c$$

**Figure 6:**
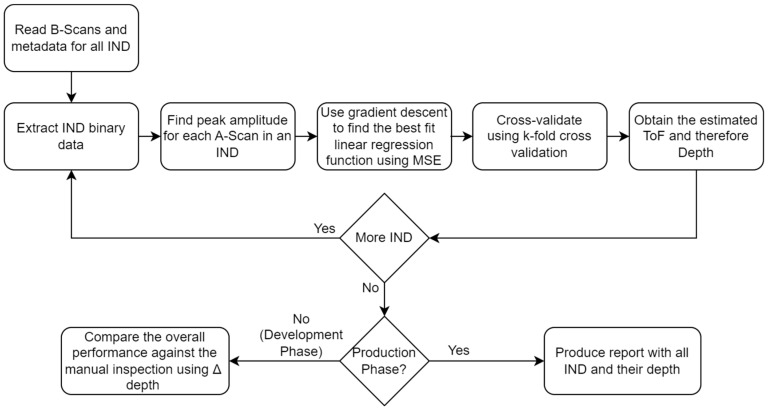
Segmented linear regression algorithm structure.

Figure 6 illustrates the flowchart for the proposed segmented linear regression method. As can be seen from the figure, the first step is to read all B-Scan files and then extract all the IND data based on the meta-data provided. Then, for each IND, a separate linear regression function is obtained using the gradient descent algorithm with the MSE loss function, as shown in Equation (3). The function utilizes the peak amplitude ToF for each A-Scan, as shown in Figure 2 and Figure 3. The function is cross-validated using k-fold cross-validation with k = 5 using a 75/25 split. Once the linear regression function is determined for that IND, the approximate ToF and depth are obtained, as shown in Equations (2) and (4). Finally, a report is produced to summarize the details for the INDs locations and depth while utilizing in production. During the development phase, the overall performance is compared against the manual inspection method by using the depth difference (Δ) between various streams to evaluate the overall performance. This is explained in detail in Section 5.

Unlike the first algorithm, which uses linear regression to approximate all ToF, the second algorithm for auto-depth measurement relies on calculating the average ToF for the region of interest (IND location ± width) while excluding outliers. The algorithm obtains the ToF for each A-Scan for the region of interest, it then calculates the standard deviation (σ) using the formula in (5), and finally, it calculates the average ToF $\left(\bar{\mathrm{ToF}}\right)$ for all points within ±σ (standard deviation) using the formula in (6). The average ToF difference is translated into IND depth by multiplying it with a constant, as previously shown in (4). This algorithm follows a similar structure to the segmented linear regression, which is shown in Figure 6, with the difference that the ToF is obtained using the formulas in Equations (5) and (6).

(5) $$\sigma =\frac{1}{N}\;\overset{N}{\underset{i=1}{\sum }}{\left(\mathrm{ToF}i-µ\right)}^{2}$$

(6) $$\bar{\mathrm{ToF}}=\frac{1}{N}\;\overset{N}{\underset{i=1}{\sum }}\mathrm{ToF}i$$

As can be seen from the formulas above, both methods use different mathematical models. Both methods can be compared to the model in [20], which presents a deep-learning solution for the detection of flaws in CANDU pressure tubes in UT scans. The solution relied on a custom-trained convolutional neural network (CNN) to perform the automation using computer vision. The focus was on locating flaws without performing depth measurements. The solution grouped 100 A-Scans and truncated a significant amount of data that was not valuable in decision-making. The inputs represented scanning points with a resolution of 1 mm/2°; these points were passed to a custom-developed and trained CNN network to conduct binary classification on each input point to detect possible flaws. This solution achieved an accuracy of 93.15%, a sensitivity of 91.17%, and a specificity of 93.72% based on the 10,000 cross-validation samples. Nevertheless, given that pressure tube INDs are, in general, larger than the scanning resolution of 1 mm/2°, detecting a portion of a flaw using one scanning point is sufficient to locate the IND. The method was tested on 18 B-Scans, where all flaws were at least partially detected [20].

## 5. Results

**Figure 7:**
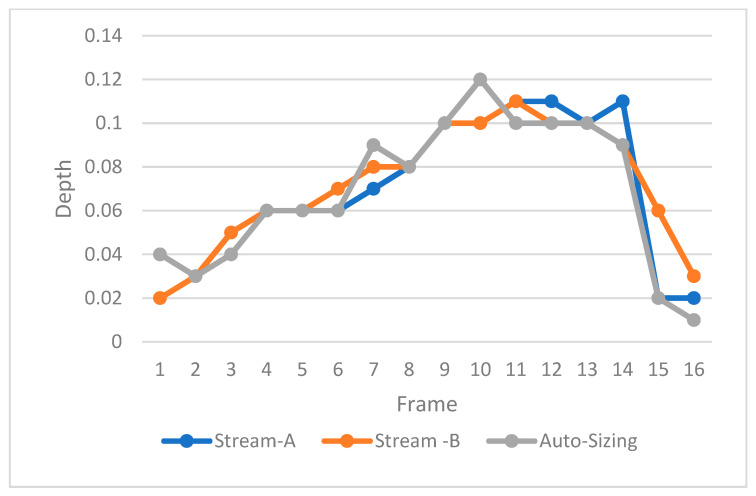
A sample for the auto sizing of one IND with 16 A-Scans.

**Figure 8:**
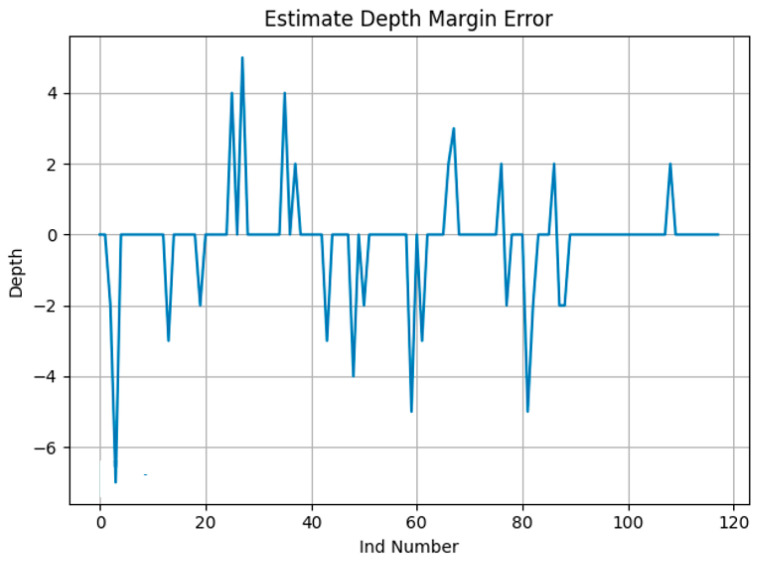
Depth difference between the auto-sizing algorithm using linear regression and the average of streams A and B for an outage data.

A total of 118 INDs were automatically extracted from the data provided by a nuclear operator. The depth of each reported frame in each IND was calculated using linear regression. Furthermore, the depth delta between ‘Stream A’ and ‘Stream B’ was calculated. The testing included a total of 3015 A-Scans. Table 1 presents the relative accuracy of the two proposed algorithms based on all A-Scans in each IND. These results are relative since both Stream A and Steam B are also subject to uncertainty. On the other hand, Table 2 presents the relative accuracy when only considering the A-Scan with the maximum depth in each IND. Table 1 and Table 2 utilize the average depth difference (delta Δ) between ‘Stream A’ and ‘Stream B’ when comparing both streams against the proposed auto-sizing algorithms. Figure 7 shows an example of depth measurement for one IND with 16 A-Scans, while Figure 8 illustrates the depth difference between the auto sizing with linear regression compared to the average depth of streams A and B. This is based on the A-Scans with the maximum depth in each IND, across a total of 118 INDs representing data from one outage. The figure shows the cases when the depth was greater than 0.01 mm.

Table 3 summarizes the overall performance based on the A-Scans with the maximum depth in each IND. As can be seen in the table, by using a linear regression of 80.51%, INDs achieve a maximum depth that is greater than one stream but less than the other stream, or is 0.01 mm higher or lower than both streams. This is based on a total of 118 INDs. Furthermore, the table demonstrates that both algorithms achieved very good relative accuracy. Nevertheless, the linear regression model showed superior performance. Additionally, an attempt was made to merge both algorithms to see if there would be any advantage, but this did not lead to any noticeable improvement over using linear regression alone. Overall, the presented results show real potential for the linear regression algorithm to be used in production to automate the detection and sizing of CANDU pressure tube flaws. The achieved average delta Δ using the automated depth measurements is reasonably close to the average delta Δ between ‘Stream A’ and ‘Stream B’. Table 3 presents three cases for depth auto-sizing. In the first case, the auto-sizing depth plus or minus 0.01 mm is greater than one stream, but is less than the other stream. In the second case, the auto-sizing depth plus 0.01 mm is higher than both streams. The third case is when the auto-sizing depth minus 0.01 is lower than both streams.

Unlike the previously proposed deep learning method, which is essentially a binary classifier to locate flaws, the proposed methods in this paper provide an exact prediction for the depth of each point in a UT scan. Therefore, the relative accuracy was measured by comparing against the average depth difference (delta Δ) between ‘Stream A’ and ‘Stream B’, as presented in Table 1, Table 2 and Table 3 in the next section. The deterministic proposed methods use a resolution of 0.1 mm/0.2°, as it calculates the depth of each single A-Scan, unlike the previously proposed deep learning method [20], which uses a much lower resolution of 1 mm/2. Additionally, these methods can calculate the depth of each flaw, while the previously proposed deep learning method is a binary classifier that can identify flaw locations in a B-Scan file. Given the large differences between the two proposed deterministic methods in this paper, and the computer vision method which is proposed in [20], the two automation solutions can be considered independent and can be used in parallel to automate the process of locating flaws. Such redundancy is highly desirable in the nuclear industry due to the sensitivity of the target application, as illustrated by the fact that manual inspection relies on two streams; therefore, any future adoption of an automation method would benefit from the availability of two or more independent techniques.

## 6. Conclusions

This paper presented solutions for automated depth measurements of flaws within CANDU pressure tube UT B-scans. Two algorithms for automating the analysis of B-scan Type-A and Type-D files using the 20 MHz NB were proposed. One algorithm used segmented linear regression, while the other used the ToF mean for the data between ±σ (standard deviation). The relative accuracy of the proposed algorithms was compared against the average depth difference (delta Δ) between the previous manual inspection by two analysts referred to as ‘Stream A’ and ‘Stream B’. Both algorithms achieved excellent relative accuracies, as presented in the results, with the segmented linear regression algorithm being superior in terms of relative accuracy. While the proposed algorithms for this technique described are CANDU specific, providing solutions for the auto-sizing of depth sizing of ID flaws can be used for a wide range of industrial applications that use NDE for piping/tubing inspection.

## Figures and Tables

**Table 1:** Auto depth measurement relative accuracy based on all A-scans in each IND.

Streams/Algorithm	Average Δ Depth (mm) Using All A-Scans in Each IND
Stream A vs. Stream B	0.0156
Linear Regression (auto) vs. Stream A	0.0189
Linear Regression (auto) vs. Stream B	0.0180
ToF Mean (auto) vs. Stream A	0.0206
Stream A vs. Stream B	0.0156

**Table 2:** Auto depth measurement relative accuracy based on the A-scans with the maximum depth in each IND.

Streams/Algorithm	Average Δ Maximum Depth (mm) Using All A-Scans in Each IND
Stream A vs. Stream B	0.0131
Linear Regression vs. Stream A	0.0169
Linear Regression vs. Stream B	0.0156
ToF Mean (auto) vs. Stream A	0.0205
ToF Mean (auto) vs. Stream B	0.0166

**Table 3:** Auto depth measurement relative accuracy based on the A-Scans with the maximum depth in each IND.

Case	Total INDs (Linear Reg.)	INDs Percentage (Linear Reg.)	Total INDs (ToF Mean)	INDs Percentage (ToF Mean)
#1	95	80.51%	77	65.81%
#2	9	7.63%	31	26.49%
#3	14	11.87%	10	8.54%

Note: the auto-sizing depth is noted as (AS-D) while ‘Stream A’ depth is noted as (A-D), and ‘Stream-B’ depth is noted as (B-D). Case #1: (AS-D ± 0.01 mm ≥ A-D and AS-D ± 0.01 mm < B-D) or (AS-D ± 0.01 mm ≥ A-B and AS-D ± 0.01 mm < B-A); Case #2: (AS-D > A-D + 0.01 mm) and (AS-D > A-B + 0.01 mm); Case #3: (AS-D < A-D − 0.01 mm) and (AS-D < A-B − 0.01 mm).

## Data Availability

Not applicable.
